# Joint effects of atrial fibrillation and prothrombotic genotypes on the risk of venous thromboembolism

**DOI:** 10.1016/j.rpth.2025.102880

**Published:** 2025-05-08

**Authors:** Erin Mathiesen Hald, Maja–Lisa Løchen, Kristian Hveem, Mary Cushman, Sigrid K. Brækkan, John–Bjarne Hansen

**Affiliations:** 1 Thrombosis Research Group, Department of Clinical Medicine, UiT The Arctic University of North Norway, Tromsø, Norway; 2 Division of Internal Medicine, University Hospital of Norway, Tromsø, Norway; 3 Epidemiology of Chronic Diseases Research Group, Department of Community Medicine, UiT The Arctic University of Norway, Tromsø, Norway; 4 Department of Public Health and Nursing, HUNT Center for Molecular and Clinical Epidemiology, Norwegian University of Science and Technology, Trondheim, Norway; 5 Department of Public Health and Nursing, HUNT Research Center, Norwegian University of Science and Technology, Levanger, Norway; 6 Levanger Hospital, Nord–Trøndelag Hospital Trust, Levanger, Norway; 7 Department of Medicine, Larner College of Medicine at the University of Vermont, Burlington, Vermont, USA

**Keywords:** atrial fibrillation, epidemiology, polymorphism, single nucleotide, prospective studies, venous thromboembolism

## Abstract

**Background:**

Atrial fibrillation (AF) is a risk factor for venous thromboembolism (VTE), but the role of common prothrombotic gene variants in this relationship is unknown.

**Objectives:**

We investigated the joint effect of prothrombotic genotypes and AF on the risk of VTE in a population-based case-cohort.

**Methods:**

Incident VTE cases (*n* = 1458) and a subcohort (*n* = 14,526) randomly sampled from the Tromsø (1994-2012) and Trøndelag Health (1995-2008) cohort studies were included. DNA was genotyped for rs8176719 (ABO), rs6025 (factor V Leiden [FVL]), rs1799963 (prothrombin), rs2066865 (fibrinogen gamma gene), and rs2036914 (F11). Hazard ratios (HRs) with 95% CIs for VTE were estimated by individual single-nucleotide polymorphisms and categories of a genetic risk score (0-1, 2, 3, 4, and ≥5 risk alleles) in subjects with and without AF.

**Results:**

Over a median 12.3 years follow-up, 1421 participants were diagnosed with AF, of whom 139 developed subsequent VTE. Overall, participants with AF had a 1.7-fold increased risk of VTE (HR, 1.73; 95% CI, 1.43-2.08). Among those with AF, ≥1 risk allele of FVL was associated with 1.9-fold higher VTE risk (HR, 1.89; 95% CI, 1.13-3.17) compared with 0 risk alleles. None of the other single-nucleotide polymorphisms increased the risk. In participants without AF, the VTE risk increased linearly with increasing genetic risk score. No such association was found for those with AF.

**Conclusion:**

We confirmed that AF is a risk factor for VTE and showed that this relationship was augmented for carriers of FVL. Other common prothrombotic genotypes do not add additional risk of VTE to that induced by AF alone.

## Essentials

• We studied prothrombotic SNPs, AF, and risk of VTE.
• In a large case-cohort, 5 genotypes were assessed individually and in a genetic risk score.
• FVL was the only prothrombotic SNP associated with VTE risk in AF.
• The 5-SNP genetic risk score did not add risk to the risk of VTE associated with AF.

## 1 Introduction

Over the last decade, etiologic population-based studies have implicated that atrial fibrillation (AF) is a risk factor for venous thromboembolism (VTE) [[1], [2], [3], [4]]. In the Tromsø study, there was an increased VTE risk in subjects with AF, and the association was particularly pronounced for pulmonary embolism (PE) in the first 6 months following AF diagnosis (11-fold increased risk) [1,2]. Similar findings were confirmed in the Atherosclerotic Risk in Communities cohort [3].

While VTE is a multicausal disease, genetic variants contribute significantly to VTE etiology, and several single-nucleotide polymorphisms (SNPs) associated with increased VTE risk have been identified [5,6]. De Haan et al. [7] developed a genetic risk score (GRS) based on the number of risk alleles of 5 SNPs (rs8176719 [non-O blood type] in ABO, rs6025 [factor V Leiden {FVL}] in F5, rs1799963 [prothrombin G20210A] in F2, rs2066865 in the fibrinogen gamma gene [FGG], and rs2036914 in F11) that predicted the risk of incident VTE equally well as 31 SNPs previously associated with VTE. Later studies confirmed that increasing GRS was associated with incident and recurrent VTE [8,9] and yielded a supra-additive effect on VTE risk when combined with other comorbidities, such as cancer and ischemic stroke [10,11], while healthier lifestyle could mitigate the genetic VTE risk [12].

Mechanistically, AF may cause PE by the formation of right atrial thrombi that embolize to the pulmonary circulation [13,14]. Furthermore, AF is associated with procoagulant changes that may increase VTE risk [15]. While both family studies and genome-wide association studies have demonstrated a strong genetic component for AF *per se* [16], it is less clear whether the presence of prothrombotic genotypes affects AF and AF outcomes. A higher prevalence of the G20210A prothrombin mutation was reported in AF subjects compared with healthy controls, yet in these studies, the presence of the prothrombin mutation was not associated with an increased risk of thromboembolic events (stroke, transient ischemic attack, or peripheral embolism) [17,18]. By contrast, the O blood type, which is associated with reduced VTE risk [19], was associated with fewer thrombotic events (ischemic stroke and noncerebral thromboembolism) in a study of 1170 AF patients compared with the AB blood type, while no difference was observed when compared with blood types A and B [20]. In a small case-control study, there was no difference in the prevalence of FVL and G20210A prothrombin mutation among AF patients with and without verified left atrial thrombus [21].

To the best of our knowledge, no previous study has investigated the joint impact of prothrombotic genotypes and AF on VTE risk. Thus, the aim of the present study was to investigate the combined effect of AF and the prothrombotic SNPs included in the GRS on VTE risk in a population-based case-cohort.

## 2 Methods

### 2.1 Study population

The study population was derived from 2 Norwegian population-based cohorts: the fourth survey of the Tromsø study (Tromsø 4) and the second survey of the Trøndelag Health (HUNT 2) study. Tromsø 4 was conducted in 1994 to 1995, and all inhabitants of the Tromsø municipality aged 25 years or older were invited to participate. In total, 77% (*n* = 27,158) of the eligible population participated. For HUNT 2, all inhabitants of Nord–Trøndelag County aged ≥20 years were invited to participate, and 66,140 persons attended (71%). Detailed descriptions of the studies have been published previously [22,23].

The participants in both studies were followed from the date of inclusion until a VTE event, migration, death, or the end of follow-up (December 31, 2012, for the Tromsø study, and December 31, 2008, for the Trøndelag Health [HUNT] study). Incident VTEs during follow-up were identified by searching the electronic patient registries at the University Hospital of North Norway (Tromsø 4) and Levanger, Namsos, and St. Olavs Hospitals (HUNT 2), as detailed previously [24,25]. In brief, all potential VTE cases were reviewed by trained personnel, and a VTE event was adjudicated as an outcome only when signs and symptoms of VTE were present and combined with confirmatory radiology procedures resulting in treatment initiation. An additional search of the autopsy registry was performed in the Tromsø study, and VTE cases were registered as a validated outcome when the death certificate indicated VTE as the cause of death or as a significant condition contributing to death.

**Figure 1:**
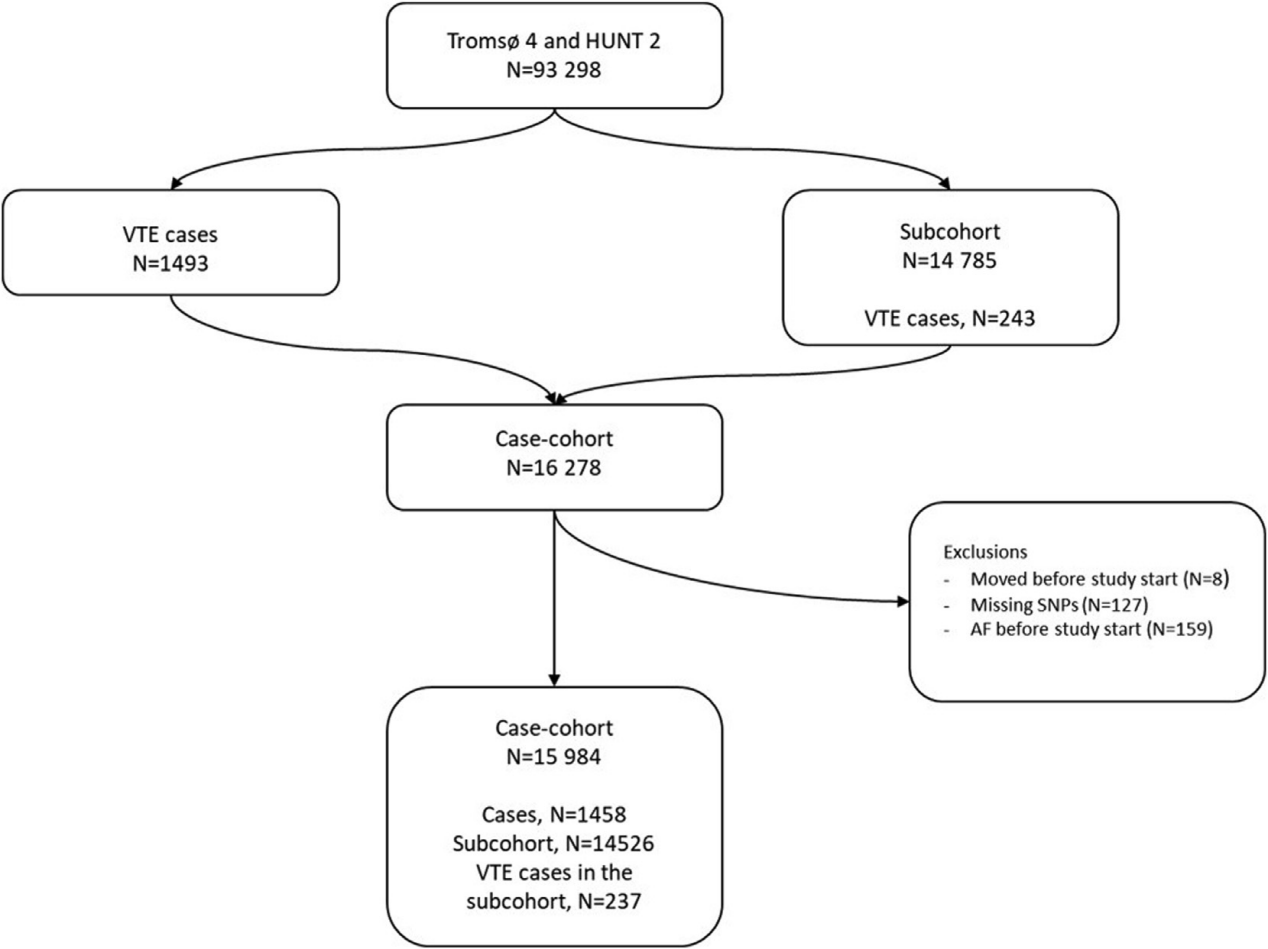
Study population assembly. Participants were recruited from the fourth survey of the Tromsø study (Tromsø 4; 1994-2012) and the second survey of the Trøndelag Health (HUNT 2; 1995-2008) study. AF, atrial fibrillation; SNP, single-nucleotide polymorphism; VTE, venous thromboembolism.

For this study, we included all participants with an incident VTE (*n* = 1493) from the Tromsø and HUNT studies as cases, and randomly sampled a subcohort (*n* = 14,785) of participants from the full parent cohorts (Figure 1). Due to the case-cohort study design, 243 of the cases were also sampled and included in the subcohort. Participants with AF prior to study enrollment (*n* = 159), participants with at least 1 missing value for the risk alleles (*n* = 127), and those not officially registered as inhabitants in Tromsø or Nord–Trøndelag at inclusion (*n* = 8) were excluded, yielding a case-cohort sample of 1458 VTE cases and 14,526 subcohort participants (Figure 1). For the SNP rs8176719 tagging non-O blood type, genotyping was missing for 12% of the subcohort (*n* = 1891). The study was approved by the Regional Committee of Medical and Health Research Ethics, and all participants provided informed written consent.

### 2.2 Prothrombotic genotypes

The SNPs included in the present study were rs8176719 (non-O blood type) in ABO, rs6025 (FVL) in F5, rs1799963 (prothrombin G20210A) in F2, rs2036914 in F11, and rs2066865 in FGG. Genotyping was performed using the Sequenom platform (Sequenom) (for rs8176719, rs6025, rs1799963, and rs2036914) and TaqMan platform (Thermo Fisher) (for rs2066865) in the Tromsø study, as detailed elsewhere [26]. In the HUNT study, genotyping was performed using the Illumina HumanCore Exome array [27].

Subjects were considered carriers of the prothrombotic risk gene if 1 or 2 risk alleles were present, with no differentiation between hetero- and homozygous carriers due to few homozygote subjects. The only genetic variant with a minor allele associated with lower VTE risk was rs2036914 (F11), and in this case, the common allele was considered the risk allele. We used the GRS conceived by de Haan et al. [7], which was created by summarizing the number of risk alleles of the 5 aforementioned SNPs.

### 2.3 Assessment of AF

In the Tromsø study, incident cases of AF were identified by searching the discharge diagnosis registry at the University Hospital of North Norway and the Norwegian Cause of Death registry provided by the Norwegian Institute of Public Health. The individual Norwegian 11-digit personal identification number allowed linking the Tromsø study participants to the diagnosis registries using the following diagnosis codes: International Classification of Diseases (ICD), Ninth Revision codes 427.0 to 427.99, and 10th Revision codes I47 and I48. Furthermore, hospital records for participants with a diagnosis of cerebrovascular or cardiovascular events but without an arrhythmia diagnosis were searched for notes on AF to ensure case completeness. The diagnosis of AF had to be documented by an electrocardiogram, and an independent end-point committee reviewed the medical records of each potential AF case and adjudicated all events [28].

Incident cases of AF in the HUNT study were identified by linking the participants’ unique Norwegian personal identification numbers to the hospital diagnosis registries in Nord–Trøndelag County using the ICD, Ninth Revision code 427.3 and 10th Revision code I48 (AF). These registries include AF diagnoses coded as primary or contributory for all inpatient and outpatient visits.

### 2.4 Statistical analysis

Statistical analyses were performed using STATA version 17.0 (Stata Corporation). Cox proportional hazards regression models were used to obtain hazard ratios (HRs) with 95% CIs for VTE by the individual SNPs or by categories of the 5-SNP score (ie, 0-1, 2, 3, 4, and ≥5 risk alleles) in participants with and without AF. AF was included as a time-varying exposure variable using 2 observation periods per study participant in those who developed AF. None of the participants had AF at study entry, and the variable was updated in subjects who experienced AF during follow-up. Thus, all participants who developed AF during follow-up contributed to nonexposed person time from the date of enrollment until AF diagnosis, and to AF-exposed person time from that date onward. In analyses estimating the joint risk by AF and the individual SNPs, observation periods without AF in participants with no risk alleles were used as the reference category. When assessing the joint effect of AF and the 5-SNP score, observation periods without AF in participants with 0 to 1 risk alleles were set as the reference category. We also performed supplementary Cox regression analyses restricted to AF exposure to further assess the impact of the individual SNPs and the increasing GRS (those with AF and no risk alleles served as reference). Chronological age was used as time scale in the Cox models, with the participants’ age at study enrollment defined as entry time in the study, and age at the censoring event (VTE, death, migration, or study end) as exit time in the study (in addition, for those who developed AF, exit time of the first observation period and entry time of the second observation period was the age at AF). All analyses were adjusted for age (as time scale), body mass index, and sex. In the present study, sex refers to the biological attributes that differentiate individuals as male or female, and was ascertained by the participants' unique personal identification number at inclusion in the Tromsø and HUNT studies. The proportional hazard assumption was tested using Schoenfeld residuals and was not violated. The presence of biological interaction between the individual SNPs and AF on risk of VTE was assessed by calculating the relative excess risk due to interaction (RERI). A RERI >0 suggests positive interaction, ie, the effect of the joint exposure to 2 risk factors is greater than the sum of the separate effects [29].

## 3 Results

**Figure 2:**
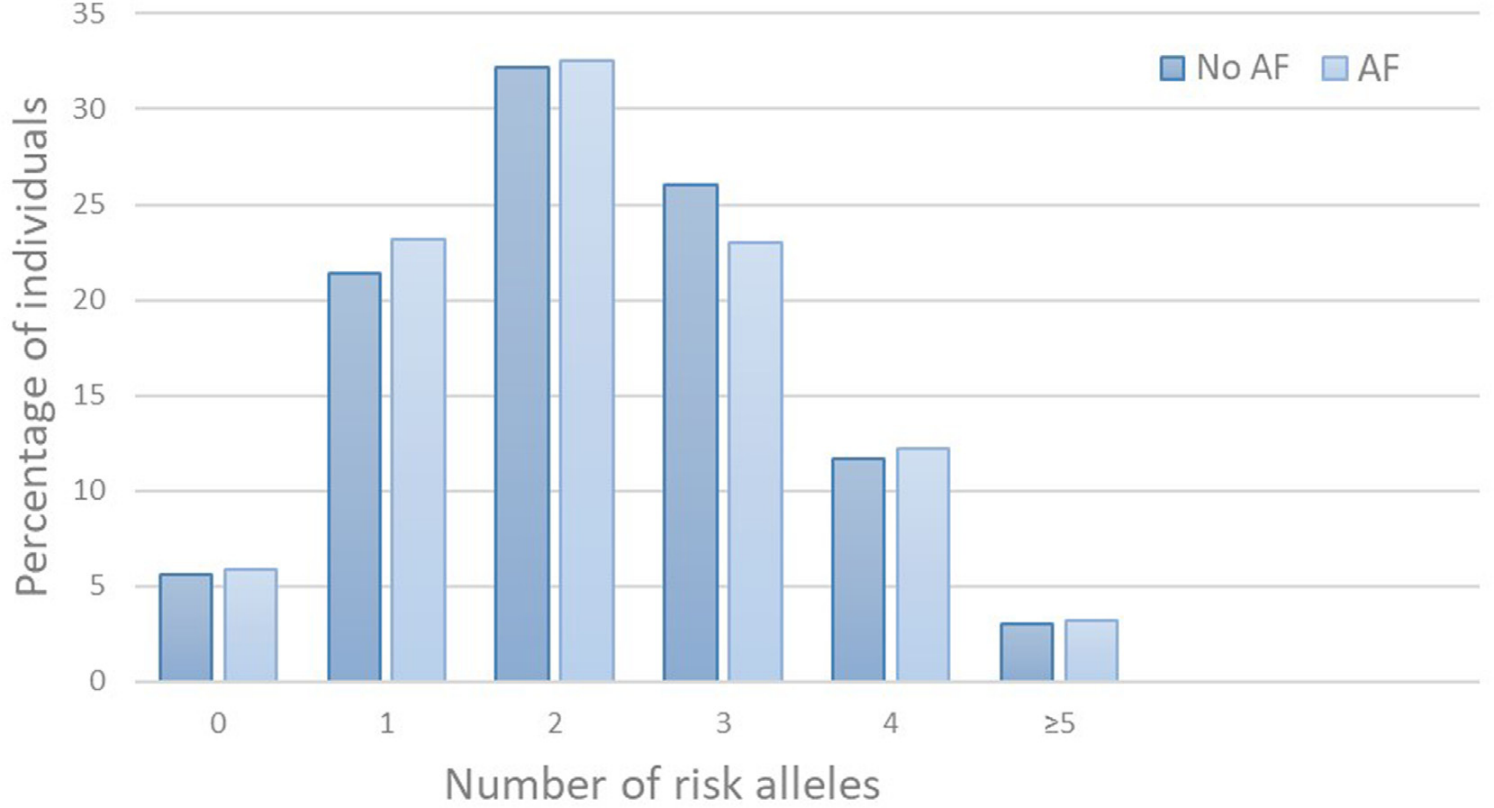
Distribution (%) of individuals across number of risk alleles of 5 prothrombotic genotypes in study participants with and without atrial fibrillation (AF).

During a median follow-up of 12.3 years (range, 9 days-18.3 years), 1421 study participants were diagnosed with AF, of whom 139 (9.8%) subsequently developed VTE. Baseline characteristics of the study population by AF status are shown in Table 1. Participants diagnosed with AF during follow-up were considerably older (mean age 67 vs 51 years) and more frequently male (52.9% vs 45.8%) than those who did not develop AF. Compared with AF-free participants, participants with AF had a less favorable cardiovascular risk profile, including a higher proportion of hypertension, physical inactivity, self-reported diabetes, previous myocardial infarction, and higher cholesterol measures. The proportion of participants having ≥1 risk allele(s) for the studied SNPs was similar between those with and without AF (Table 1). The GRS risk allele distribution was comparable for those with and without AF (Figure 2), with a median of 2 risk alleles for both groups (range, 0-7).

**Table 1:** Baseline characteristics of the study population with and without atrial fibrillation.

Baseline characteristics	No AF (*n* = 14,563)	AF (*n* = 1421)
Age (y)	51 (±16)	67 (±10)
Sex (male)	45.8 (6672)	52.9 (752)
BMI (kg/m^2^)	26.2 (±4.1)	27.4 (±4.4)
Total cholesterol (mmol/L)	6.1 (±1.4)	6.6 (±1.3)
Systolic blood pressure (mm Hg)	138 (±21)	153 (±25)
Diastolic blood pressure (mm Hg)	80 (±12)	86 (±14)
Hypertension^a^	44.8 (6535)	74.3 (1056)
Smoking^b^	31.0 (4502)	24.2 (342)
Physical activity^c^	30.2 (3416)	20.2 (222)
Self-reported diabetes mellitus	2.8 (405)	7.2 (101)
History of myocardial infarction	5.5 (795)	21.9 (311)
Rs817619 (*ABO*)^d^^,^^e^	62.6 (8132)	62.6 (695)
rs6025 (*F5*)^e^	7.4 (1075)	8.4 (120)
rs1799963 (*F2*)^e^	1.5 (212)	1.5 (21)
rs2066865 (*FGG*)^e^	42.9 (6250)	44.6 (634)
rs2036914 (*F11*)^e^	74.5 (10,853)	74.0 (1051)

Values are % (*n*) or mean (±SD). Genes related to the single-nucleotide polymorphisms are depicted between parentheses.

AF, atrial fibrillation; BMI, body mass index.

a Mean systolic/diastolic blood pressure ≥ 140/≥ 90 mm Hg or current use of antihypertensive drugs.

b Self-reported daily smoking, yes/no. Information on physical activity was missing for 22% of the total case-cohort.

c ≥1 Hour of moderate or hard physical activity per week, yes/no.

d ABO genotyping was missing for 12% of the total case-cohort.

e Percentage of participants with ≥1 risk allele.

The clinical characteristics of the VTE events by AF status are presented in Table 2. PE was slightly more frequent among AF participants than those without AF (47.6% vs 38.9%), and those with AF had fewer cancer-related VTE events than the non-AF group (16.0% and 20.6%, respectively).

**Table 2:** Clinical characteristics of venous thromboembolism events: the Tromsø and Trøndelag Health studies.

Clinical characteristics, % (*n*)	No AF (*n* = 1189)	AF (*n* = 269)
• DVT	61.1 (726)	52.4 (141)
• PE	38.9 (463)	47.6 (128)
• Unprovoked	43.6 (519)	45.5 (122)
• Provoked^a^	56.4 (670)	54.5 (146)
o Cancer-related	20.6 (245)	16.0 (43)

Provoking factors: major surgery, trauma, or an acute medical condition within 8 weeks prior to the event; active cancer at the time of the event; and marked immobilization (bed rest for longer than 3 days, confinement to a wheelchair, or long-distance travel exceeding 4 hours within the last 14 days prior to the event) were considered provoking factors.

AF, atrial fibrillation; DVT, deep vein thrombosis; PE, pulmonary embolism.

a Information on provoking factors was missing for 1 venous thromboembolism event.

Overall, participants with AF had a 1.7-fold increased risk of VTE compared with participants without AF (HR, 1.73; 95% CI, 1.43-2.08). The HRs for VTE by categories of the individual SNPs and AF status are shown in Table 3. Participants with non-O blood type without AF had a 1.4-fold higher risk of VTE (HR, 1.41; 95% CI, 1.25-1.58) compared with those with blood type O without AF. In participants with non-O blood type and AF, the risk was 2.6-fold higher (HR, 2.56; 95% CI, 2.02-3.25) than the reference category, while those with AF without non-O blood type had a 2.3-fold (HR, 2.29; 95% CI, 1.68-3.12) increased VTE risk compared with the reference category. Having ≥1 risk allele of FVL was associated with a 2.5-fold (HR, 2.48; 95% CI, 2.14-2.88) increased VTE risk in participants without AF, and a 3.5-fold (HR, 3.46; 95% CI, 2.14-5.60) increased risk in participants with AF compared with those without either risk factor. Calculation of the RERI for FVL and AF revealed that the combined risk was only slightly higher than the sum of the individual components, with a RERI of 0.2. Accordingly, in those with AF, having ≥1 FVL risk allele increased the VTE risk by 1.9-fold compared with those without FVL (HR, 1.89; 95% CI, 1.13-3.17). For the F2 SNP, risk estimates for VTE were similar for participants with and without AF. We observed no significant association between the FGG SNP and VTE nor between the F11 SNP and VTE in participants without AF (HR, 1.11; 95% CI, 1.00-1.24 and HR, 1.00; 95% CI, 0.89-1.14, respectively). In those with AF, having either the FGG or F11 risk allele(s) did not add significant risk beyond AF alone (Table 3).

**Table 3:** Hazard ratios with 95% CIs of venous thromboembolism according to individual single-nucleotide polymorphisms: the Tromsø and Trøndelag Health studies.

SNP (gene)	Risk alleles	Events	HR (95% CI)^a^	HR (95% CI)^b^
***rs8176719 (ABO)***^c^				
No AF	0	409	Ref	
	≥1	918	1.41 (1.25-1.58)	
AF	0	46	2.29 (1.68-3.12)	Ref
	≥1	85	2.56 (2.02-3.25)	1.14 (0.79-1.64)
**rs6025 (*F5*)**				
No AF	0	1119	Ref	
	≥1	208	2.48 (2.14-2.88)	
AF	0	114	1.78 (1.46-2.17)	Ref
	≥1	17	3.46 (2.14-5.60)	1.89 (1.13-3.17)
**rs1799963 (*F2*)**				
No AF	0	1298	Ref	
	≥1	29	1.63 (1.13-2.35)	
AF	0	129	1.74 (1.44-2.10)	Ref
	≥1	2	1.77 (0.44-7.14)	1.00 (0.24-4.21)
**rs2066865 (*FGG*)**				
No AF	0	721	Ref	
	≥1	606	1.11 (1.00-1.24)	
AF	0	69	1.66 (1.29-2.14)	Ref
	≥1	62	2.02 (1.55-2.63)	1.26 (0.89-1.77)
**rs2036914 (*F11*)**				
No AF	0	325	Ref	
	≥1	1002	1.00 (0.89-1.14)	
AF	0	39	1.99 (1.42-2.79)	Ref
	≥1	92	1.64 (1.29-2.08)	0.81 (0.56-1.19)

AF, atrial fibrillation; HR, hazard ratio; Ref, reference; SNP, single-nucleotide polymorphism.

a All HRs are adjusted for age (as time scale), sex, and body mass index.

b Analysis restricted to those with AF exposure.

c ABO genotyping was missing for 12% of the total case-cohort.

For participants without AF, the VTE risk increased across increasing categories of risk alleles in the GRS (Table 4). Having ≥5 risk alleles was associated with a 2.4-fold increased risk of VTE compared with having 0 to 1 risk alleles (HR, 2.38; 95% CI, 1.85-3.06). In participants with AF, there was no linear increase in VTE risk across categories of the 5-SNP score. However, having ≥5 risk alleles and AF was associated with the highest VTE risk (HR, 4.23; 95% CI, 2.09-8.48), though the absolute number of VTE events in this category was low (Table 3). As we found null associations for VTE risk regardless of AF status for F11 and FGG, we performed supplementary analyses omitting these SNPs from the GRS, and the overall associations remained unchanged (data not changed).

**Table 4:** Hazard ratios of venous thromboembolism by categories of the genetic risk score.

GRS^c^	Risk alleles	Events	HR (95% CI)^a^	HR (95% CI)^b^
No AF	0-1	250	Ref	
	2	367	1.16 (0.99-1.36)	
	3	407	1.56 (1.33-1.83)	
	4	222	1.73 (1.44-2.07)	
	≥5	81	2.38 (1.85-3.06)	
AF	0-1	33	2.49 (1.72-3.59)	Ref
	2	36	2.41 (1.69-3.43)	0.98 (0.61-1.58)
	3	36	2.77 (1.94-3.94)	1.12 (0.70-1.81)
	4	18	2.75 (1.70-4.45)	1.11 (0.62-1.99)
	≥5	8	4.23 (2.09-8.58)	1.71 (0.79-3.73)

AF, atrial fibrillation; GRS, genetic risk score; HR, hazard ratio; Ref, reference.

a Adjusted for age (as a time scale), sex, and body mass index.

b Analysis restricted to those with AF exposure.

c ABO genotyping was missing for 12% of the total case-cohort.

Considering PE and deep vein thrombosis (DVT) separately, the association between AF and a subsequent thrombotic event was stronger for PE than for DVT (Supplementary Tables S1 and S2). When analyzing the individual SNPs, the risk estimates for PE were not significantly higher for those with AF and ≥1 risk allele than for those with AF alone. In contrast, persons with ≥1 risk allele in FVL and AF had a 3.4-fold higher risk of DVT compared with persons with AF without FVL (HR, 3.37; 95% CI, 1.81-6.30; Supplementary Table S2).

## 4 Discussion

In the present study, we examined the joint impact of 5 prothrombotic genotypes and AF on the risk of VTE in a population-based case-cohort. In agreement with previous findings, participants with AF had a pronounced risk of incident VTE. Among those with AF, carriers of the FVL risk allele(s) had an increased VTE risk compared with noncarriers. The presence of any of the other 4 prothrombotic SNPs did not add to the VTE risk associated with AF. In participants without AF, the risk of VTE increased across categories of the GRS, whereas no such association was found for those with AF.

An increased VTE risk by AF is in agreement with previous findings from large observational studies [[1], [2], [3],^30^,^31^]. In studies including the full Tromsø study cohort, participants with AF had a 1.4-fold long-term risk of future VTE [1] and a >10-fold increased risk of PE in the 6-month period after AF diagnosis [2]. The excess risk of VTE by AF appeared irrespective of the intermediate development of ischemic stroke [2,30] and remained after accounting for cardiovascular risk factors [2,3]. In the present study, most of the prothrombotic SNPs did not add to VTE risk in AF, suggesting that mechanisms other than the procoagulant milieu that these SNPs contribute to may explain VTE development in AF. As the VTE risk is especially pronounced during the first months following AF diagnosis and diminishes over time [1,2,30,31], the association may partly be explained by factors related to the onset of AF, such as heart failure, transient hypercoagulability, hospitalization, immobilization, and concurrent infection [32].

Our findings confirmed recognized associations for the studied SNPs and GRS as risk factors for VTE *per se* [5,6,33]. We additionally found that the combined effect of AF and the rs6025 (FVL) SNP yielded a slightly higher risk than the sum of the individual components. In subjects with AF, those with ≥1 risk allele in FVL had 1.9-fold higher VTE risk compared with those with no risk allele. While we are not aware of other studies assessing FVL as a VTE risk factor in AF, a few studies have previously examined the impact of FVL on arterial thrombosis risk in AF patients. In a nested case-control study of adult patients with AF, Go et al. [34] found a nonsignificantly higher prevalence of heterozygous FVL carriers among ischemic stroke cases than controls. Berge et al. [35] found no association between FVL and first–ever stroke in a small hospital-based case-control study of patients with AF, and there was no observed difference in the prevalence of FVL among AF patients with and without an arterial thromboembolic event in a case-control study from Italy [18]. In the present study, FVL was associated with a 3-fold increased risk of DVT in patients with AF, while no increased risk was observed for PE. This differential impact on the 2 outcomes has been identified previously as the so-called “FVL paradox” [36]. The mechanisms underlying this phenomenon are not fully understood [36,37]. In a mouse model, presence of the FVL mutation was associated with higher DVT stability and lower PE rates, suggesting that FVL carriers have lower embolization risk [38].

Our study has several strengths, including the prospective cohort design and long-term follow-up. Both the Tromsø and the HUNT studies had high participation rates across age groups, ensuring a source cohort representative of the general population, and the VTE events were rigorously validated. Some limitations also merit consideration. For the participants recruited from the HUNT study, information on the exposure variable, AF, was derived from discharge diagnosis registries. While a population-based Swedish study found that electronic health data identified 93.2% of AF cases [39], a validation study of the HUNT 3 population found the sensitivity and specificity of a hospital discharge diagnosis of AF to be 73.7% and 99.7%, respectively [40]. Consequently, the use of hospital-diagnosed AF in the present analyses likely underestimates the true AF incidence in the study population, which, in turn, may underestimate the association between AF and VTE. The diagnosis of AF in the present study does not account for the possibility of some participants reestablishing sinus rhythm during follow-up. Nevertheless, as recurrence rates after a first AF episode are as high as 90% [41], significant misclassification bias is unlikely. We also lacked information on anticoagulation use during follow-up. The initiation of anticoagulant treatment after AF diagnosis may effectively reduce VTE risk and thereby dilute additional effect of the prothrombotic genotypes. Specific information on race and ethnicity of the study participants was not available for the included surveys of the Tromsø and the HUNT studies, but the source population at inclusion was predominantly Caucasian subjects, which may limit the generalizability of our findings to other ethnic groups. Finally, subgroup analyses are limited by few VTE events in the low-prevalence SNP groups and must be interpreted with caution.

In conclusion, we confirmed that the occurrence of AF is associated with an increased risk of incident VTE. Except for FVL, the combined effect of AF and prothrombotic genotypes, either as individual SNPs or as a GRS, did not yield any supra-additive effect on VTE.
