# Supplementary material for: Joint effects of atrial fibrillation and prothrombotic genotypes on the risk of venous thromboembolism
Source: Res Pract Thromb Haemost. 2025 May 8;9(4):102880. doi: 10.1016/j.rpth.2025.102880 (PMC12272126; doi:10.1016/j.rpth.2025.102880)
Supplement: Supplementary Tables 1 and 2 [file mmc1.docx]

**Supplementary table S1.** Hazard ratios of pulmonary embolism for individual single nucleotide polymorphisms. The Tromsø and the HUNT studies

|  | **Risk Alleles** | **Events** | **HR (95% CI) *** | **HR (95% CI)** † |
| --- | --- | --- | --- | --- |
| **SNP (Gene)** |  |  |  |  |
| **rs8176719 (*ABO*)** *‡* |  |  |  |  |
| No atrial fibrillation | 0 | 170 | Ref |  |
|  | ≥1 | 353 | 1.31 (1.09-1.57) |  |
| Atrial fibrillation | 0 | 21 | 2.24 (1.52-3.81) | Ref |
|  | ≥1 | 47 | 3.24 (2.32-4.51) | 1.30 (0.78-2.18) |
| **rs6025 (*F5*)** |  |  |  |  |
| No atrial fibrillation | 0 | 464 | Ref |  |
|  | ≥1 | 59 | 1.69 (1.29-2.22) |  |
| Atrial fibrillation | 0 | 64 | 2.29 (1.74-3.00) | Ref |
|  | ≥1 | 4 | 1.86 (0.69-5.00) | 0.78 (0.28-2.16) |
| **rs1799963 (*F2*)** |  |  |  |  |
| No atrial fibrillation | 0 | 510 | Ref |  |
|  | ≥1 | 13 | 1.88 (1.08-3.26) |  |
| Atrial fibrillation | 0 | 66 | 2.14 (1.63-2.80) | Ref |
|  | ≥1 | 2 | 4.69 (1.15-19.01) | 2.56 (0.62-10.52) |
| **rs2066865 (*FGG*)** |  |  |  |  |
| No atrial fibrillation | 0 | 292 | Ref |  |
|  | ≥1 | 231 | 1.05 (0.88-1.25) |  |
| Atrial fibrillation | 0 | 34 | 1.91 (1.33-2.75) | Ref |
|  | ≥1 | 34 | 2.59 (1.80-3.73) | 1.36 (0.84-2.19) |
| **rs2036914 (*F11*)** |  |  |  |  |
| No atrial fibrillation | 0 | 125 | Ref |  |
|  | ≥1 | 398 | 1.03 (0.85-1.26) |  |
| Atrial fibrillation | 0 | 22 | 2.79 (1.76-4.42) | Ref |
|  | ≥1 | 46 | 2.01 (1.42-2.84) | 0.69 (0.42-1.16) |

*All HRs adjusted for age (as time scale), sex and BMI

†Analysis restricted to those with AF exposure

‡ABO genotyping was missing for 12% of the total case-cohort

**Supplementary table S2.** Hazard Ratios of deep vein thrombosis for individual single nucleotide polymorphisms. The Tromsø and the HUNT studies

|  | **Risk Alleles** | **Events** | **HR (95% CI) *** | **HR (95% CI)** † |
| --- | --- | --- | --- | --- |
| **SNP (Gene)** |  |  |  |  |
| ***rs8176719 (ABO)*** *‡* |  |  |  |  |
| No atrial fibrillation | 0 | 239 | Ref |  |
|  | ≥1 | 565 | 1.48 (1.27-1.72) |  |
| Atrial fibrillation | 0 | 25 | 2.20 (1.45-3.34) | Ref |
|  | ≥1 | 38 | 2.03 (1-44-2.88) | 1.00 (0.59-1.67) |
| **rs6025 (*F5*)** |  |  |  |  |
| No atrial fibrillation | 0 | 655 | Ref |  |
|  | ≥1 | 149 | 3.04 (2.55-3.63) |  |
| Atrial fibrillation | 0 | 50 | 1.38 (1.03-1.85) | Ref |
|  | ≥1 | 13 | 4.70 (2.70-8.16) | 3.37 (1.81-6.30) |
| **rs1799963 (*F2*)** |  |  |  |  |
| No atrial fibrillation | 0 | 788 | Ref |  |
|  | ≥1 | 16 | 1.47 (0.90-2.41) |  |
| Atrial fibrillation | 0 | 63 | 1.45 (1.17-1.90) | Ref |
|  | ≥1 | 0 | - | - |
| **rs2066865 (*FGG*)** |  |  |  |  |
| No atrial fibrillation | 0 | 429 | Ref |  |
|  | ≥1 | 375 | 1.15 (1.01-1.33) |  |
| Atrial fibrillation | 0 | 35 | 1.47 (1.03-2.09) | Ref |
|  | ≥1 | 28 | 1.58 (1.07-2.34) | 1.16 (0.70-1.91) |
| **rs2036914 (*F11*)** |  |  |  |  |
| No atrial fibrillation | 0 | 200 | Ref |  |
|  | ≥1 | 604 | 0.98 (0.83-1.15) |  |
| Atrial fibrillation | 0 | 17 | 1.45 (0.88-2.40) | Ref |
|  | ≥1 | 46 | 1.39 (1.00-1.93) | 0.97 (0.55-1.70) |

*All HRs adjusted for age (as time scale), sex and BMI

†Analysis restricted to those with AF exposure

‡ABO genotyping was missing for 12% of the total case-cohort
